# Longitudinal changes in Mediterranean diet adherence and perceived benefits and barriers to its consumption in US university students

**DOI:** 10.3389/fnut.2024.1405369

**Published:** 2024-07-02

**Authors:** Serhat Yildiz, Patrick Downing, Caroline J. Knight, Andrew D. Frugé, Michael W. Greene

**Affiliations:** ^1^Department of Nutritional Sciences, Auburn University, Auburn, AL, United States; ^2^College of Nursing, Auburn University, Auburn, AL, United States

**Keywords:** Mediterranean diet, adherence, barriers and benefits, university students, COVID-19

## Abstract

**Objective:**

The Dietary Guidelines for Americans has recommended consumption of a Mediterranean diet (MD) for overall health and wellbeing, and the US News & World Report has ranked the MD as the top diet overall for the past six consecutive years. However, it is uncertain if university students in the United States (US) have increased their adoption of this dietary approach over these past six years.

**Design:**

Longitudinal cross-sectional survey conducted in three cohorts (2018, 2020, 2022) utilizing regression models to assess MD Adherence and other relevant outcomes variables.

**Setting:**

University in the southern US.

**Participants:**

Students (*n* = 761) enrolled in undergraduate introductory nutrition course.

**Results:**

Survey respondents were 83% female, 91% white, and 97% ages 18–24. Predictors of MD adherence were older age, female gender, and health-related qualifications. MD adherence was lowest in 2022. The 2022 group perceived less MD health benefits, weight loss, ethical concerns, natural content, and sensory appeal compared to the 2018 group. During the COVID-19 pandemic, changes in eating behavior were examined in the 2020 and 2022 groups. We observed that participants in the 2022 group had a greater frequency of snacking and a lower frequency of eating out compared to 2020 group.

**Conclusion:**

MD adherence did not increase over time in US university students. These findings underscore the need for targeted interventions and education to promote healthier eating habits in university students.

## 1 Introduction

The Mediterranean Diet (MD) was initially defined as the eating habits of people living in areas surrounding the Mediterranean Sea where olive trees are grown (1). These areas include countries such as Algeria, Bosnia, Croatia, Cyprus, Egypt, France, Gibraltar, Greece, Israel, Italy, Lebanon, Libya, Malta, Monaco, Montenegro, Morocco, Palestine, Slovenia, Spain, Syria, Tunisia, and Turkey (1). The MD emphasizes a diet high in whole grains, legumes, nuts and seeds, fruits and vegetables, olive oil, poultry, and fish (2). It also advocates for a low intake of sweets, red meat, and meat products; wine consumption is moderate, respects social beliefs, and is preferably consumed during meals (2).

Consumption of the MD is associated with health benefits. The landmark Seven Countries Study (3) and other observational and ecological studies have demonstrated the favorable effects of the MD on reducing the risk of cardiovascular disease, several types of cancers, Alzheimer’s disease, Parkinson’s disease, obesity, stroke and hypertension (1). From a nutritional standpoint, the MD is characterized by a low consumption of saturated fats and animal proteins, and a high intake of antioxidants, fiber, monounsaturated fats, and an appropriate balance of omega-6 and omega-3 fatty acids (4). The health advantages of the MD can be attributed to the significant consumption of antioxidants, fiber, monounsaturated fats, omega-3 fatty acids, phytosterols, and probiotics (5).

Given the health benefits of the MD, a greater understanding of factors influencing MD consumption is needed. The precaution adoption process model (PAPM) is a sequential stage model that effectively explains the uptake of various health behaviors, including weight management (6). The PAPM is originated from the transtheoretical model (TTM) and focuses on the stages of change, but differs from the TTM by including only one variable and incorporating two additional stages, disengagement and rejection (7). This model involves seven distinct stages that range from ignorance of the behavior to completion of preventive action (“unaware,” “unengaged,” “deciding,” “decided no,” “decided yes,” “action,” and “maintenance”) (8). The stages of change in the PAPM are impacted by various factors, including an individual’s beliefs, prior experiences, knowledge, and perceptions of the benefits and barriers associated with the behavior (8). The perceived barriers and benefits of adopting a particular diet strongly influence an individual’s food choices and their likelihood of modifying their current diet (9). Customized nutrition education that corresponds to an individual’s stage of change can significantly improve the outcomes of behavior change (10).

The 2015–2020 (11) and 2020–2025 (12) Dietary Guidelines for Americans suggested that a Mediterranean-style diet is a healthy dietary choice for all adults in the United States. According to the US News & World Report, the MD has been ranked as the top diet overall for six consecutive years (13). Despite modern nutrition guidelines including the Mediterranean eating pattern as a recommended healthy dietary pattern, its adoption in the United States is regional (14, 15). Yet, MD adherence is less in the United States than in countries bordering the Mediterranean Sea in which the MD is a cultural heritage (16, 17). For young adults, the university phase often represents the first time in their lives when they begin to make their own choices about food and other aspects of their lives (18). Furthermore, the extent to which university students have adopted the MD between 2018 and 2023 since it was voted a top diet and recommended in the Dietary Guidelines for Americans remains uncertain. This study aimed to evaluate elements related to the MD among university students: the primary elements were the degree of adherence to the MD, the perceived barriers and benefits associated with the MD, and participants’ stage of change in regard to their adoption of the MD; while a secondary element was the impact of the COVID-19 pandemic on eating habits and adherence to the MD.

## 2 Materials and methods

### 2.1 Ethical approval and survey distribution

The institutional review board of Auburn University approved this study (IRB Protocol # 20-436 EX 2009) prior to dissemination. This survey was distributed using Qualtrics (Qualtrics, Provo, UT, USA) in 2018 from 23 August to 14 September, in 2020 from 23 August to 14 September, in 2022 from 23 August to 14 September 2022. Participant eligibility was students greater than 18 years old and enrolled in an introductory nutrition course (NTRI 2000) in the College of Human Sciences. The course is predominantly taken by female freshman and sophomore students. Each instructor in three sections of NTRI 2000 being taught in the fall semester of 2018, 2020, and 2022 recruited students enrolled in their section. The curriculum and textbook used in the course over the three survey periods did not change, and two of the three instructors participated in all three survey periods. The instructors emailed the students in their section an invitation written by the principal investigator that included a link to the survey on Qualtrics. Once the survey instrument was completed, the students were linked to a separate independent survey on Qualtrics to collect their name and section that was then reported back to the instructor. Students were provided extra credit points for participating, and the linking of surveys ensured that extra credit was provided anonymously. The response rate for completing the survey was 68%. Surveys were excluded for failing to meet the age requirement of 18 and having missing values. Surveys were also excluded if participants took less than 90 s (4.5% of the study population) to complete the survey. Based on the development and prior use of the survey instrument (15), we found that greater than 90 s was needed to complete the survey. A sensitivity analysis was performed to explore the effects of excluding the top 10% of the fastest responders (responders taking 252 s or less). A power analysis was performed using a sample size calculator for cross-sectional studies (19)^1^ based on a prior study of MD adherence in US University students (20). Assuming a difference of 0.5 points and a SD of 1.6 and a 5% significance test with 90% power, a sample size of approximately 224 participants per group was estimated.

### 2.2 Survey instrument

A previously validated survey instrument was used to assess participant adherence to the MD, their stage of change, barriers to adoption, and benefits of adoption, as well as demographic factors (15). The MD adherence component of the survey instrument used a validated 14-question Mediterranean diet adherence screener (MEDAS) (21) which has been employed to evaluate MD compliance in nations bordering the Mediterranean Sea basin as well as other parts of the world, including the southeast United States (20, 22, 23). For the stage of change assessment (PAPM), three questions were posed to participants to gauge their readiness to adopt a MD (stages of change) (8). Next, a set of 26 questions evaluating perceived benefits (weight loss, ethical concerns, sensory appeal, natural content, knowledge, familiarity, price, and mood) and 18 questions evaluating perceived barriers to the MD (health, convenience, sensory appeal, and knowledge) were utilized. A five-point Likert scale was used to score these questions. Sex, age, weight, height, ethnicity, level of education, and prior nutrition education or knowledge were determined via seven demographic and anthropometric questions. Body mass index (BMI) was determined using weight in pounds (lb) divided by height in inches (in) squared, multiplied by a conversion factor of 703. Each patient was categorized as underweight (BMI < 18.5), normal (BMI 18.5–24.9), overweight (BMI 25.0–29.9), and obese (BMI > 30.0). A question on class standing in college was included in the 2020 and 2022 survey instrument to determine whether class standing in college was a confounding variable. We included six additional questions in the 2020 and 2022 survey instrument to evaluate alterations in portion sizes, the types of food consumed, the frequency of snacking, the frequency of eating out, and the consumption of MD foods during the COVID-19 pandemic. The six addition questions were not included in the 2018 survey instrument because these questions only arose because of the COVID-19 pandemic. Exploratory factor analysis with varimax rotation was performed to assess the fit of a two-factor model. We assessed the root means the square of residuals (RMSR) to determine whether the value was close to 0. Next, the RMSEA (root mean square error of approximation) was assessed to determine whether the value was below 0.05. Finally, the Tucker-Lewis Index (TLI) was assessed to determine whether the value was over 0.9.

The full survey instrument is included in the Supplementary materials.

### 2.3 Statistical analyses

The Rx64 2022.12.0+353 software environment and RStudio were used for all data analyses (RStudio, PBC, Boston, MA, USA). The differences in total MEDAS scores between the groups were evaluated using an unadjusted and multivariable backward stepwise linear regression analysis. An unadjusted and adjusted linear regression analysis was employed to assess the variation in EBCS scores among the groups. Regression coefficient *p*-values and main effect *p*-values were reported. A type III Sum of Squares was employed to determine the main impact *p*-values. The results of the barriers and benefit questions were calculated using an unadjusted linear model, and an adjusted model incorporating all demographic variables. To determine the demographic characteristics that are the best predictors of the stage of change, a backward stepwise elimination logistic regression was used. The logistic regression model’s inclusion and retention standards were set at *p*-value. To examine variations in demographic categories between groups and participants by stage of change, Pearson’s chi-squared tests were used.

## 3 Results

**FIGURE 1:**
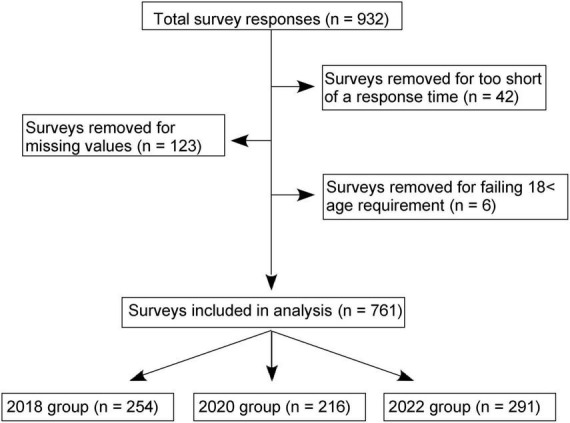
Flow chart of survey results. The survey results were gathered using Qualtrics and a total of 931 responses were obtained. After exclusions, the analysis was based on 761 surveys, with the distribution of respondents being 254 in 2018, 216 in 2020, and 291 in 2022 groups.

A total of 932 respondents completed the questionnaire (Figure 1). Surveys were excluded for: (1) taking less than 90 s to complete the survey (*n* = 42); (2) failing to meet the age requirement of 18 (*n* = 6); and (3) having missing values (*n* = 123). After exclusions, 761 valid responses were obtained. Based on the year the survey was collected, the entries were split into three groups: 2018 (*n* = 254) 2020 (*n* = 216), and 2022 (*n* = 291).

### 3.1 Demographics

We examined whether there were significant longitudinal differences in the demographics. As shown in Table 1, significant longitudinal differences (*p* < 0.05) in age, ethnicity, education, and health related qualification were found among participants. The 2022 group had the greatest proportion of the youngest (18–24 years old) participant, white participants, and high school or lower degree participants. The 2020 group had the highest percentage of participants who did not possess any qualifications related to health and nutrition. Among groups, there were no statistically significant differences in sex or BMI. We examined the class standings of participants in the 2020 and 2022 cohorts. There was no statistically significant relationship between class standings and cohorts based on Pearson’s chi-squared test (*P*-value = 0.13). However, the majority of students were sophomores in both 2020 and 2022 cohorts (Supplementary Table 1).

**TABLE 1:** Demographics of participants in the 2018, 2020, and 2022 groups.

	2018^†^		2020^†^		2022^†^		
	*N*	%	*n*	%	*n*	%	*P*-value
Sex							0.18
Male	42	16.5	46	21.3	44	15.1	
Female	212	83.5	170	78.7	247	84.9	
Age*							**0.005**
18–24	246	96.9	204	94.4	289	99.3	
25 ≥	8	3.1	12	5.6	2	0.7	
Ethnicity*							**0.006**
White	223	87.8	194	89.8	274	94.2	
Black	15	5.9	10	4.6	1	0.3	
Other	16	6.3	12	5.6	16	5.5	
Education*							**< 0.001**
High school or lower	204	80.3	191	88.4	271	93.1	
GED^††^	5	2.0	5	2.3	2	0.7	
Technical or trade certificate	1	0.4	0	0	1	0.3	
Associate degree	18	7.1	16	7.4	14	4.8	
Bachelor’s degree or higher	26	10.2	4	1.9	3	1.0	
BMI							0.070
Underweight (BMI < 18.5)	10	3.9	12	5.6	12	4.1	
Normal weight (BMI 18.5–24.9)	181	71.3	141	65.3	228	78.4	
Overweight (BMI 25–29.9)	48	18.9	46	21.3	39	13.4	
Obese (BMI > 30.0)	15	5.9	17	7.9	12	4.1	
Qualification*							**< 0.001**
Health or nutrition related qualifications	42	16.5	5	2.3	10	3.4	
No health or nutrition related qualifications	212	83.5	211	97.7	281	96.6	

*Significance across score categories by Pearson’s chi-squared test are highlighted in bold font.

^†^2018, 2020, and 2022.

^††^General Educational Development (GED) which certifies high school academic skills.

### 3.2 MD adherence by university students

An unadjusted and multivariable backward stepwise linear regression model adjusting for sex, age, health-related qualifications, and BMI was used to examine the overall MEDAS score. In the unadjusted model, for each point increase in MEDAS score in the 2018 group, a significant reduction (−0.53 ± 0.17 points, *p* = 0.001) was observed in the 2022 group, but not the 2020 group (Table 2). In the adjusted model, for each point increase in MEDAS score in the 2018 group, a significant reduction (−0.36 ± 0.17 points, *p* = 0.03) was observed in the 2022 group, but not the 2020 group, and a significant group effect (*p* = 0.020) was observed. In the adjusted model, the MEDAS score was 0.68 ± 0.18 points less in males than females (*p* < 0.001), 1.35 ± 0.42 points greater in participants aged ≥ 25 (*p* < 0.001), and 1.13 ± 0.27 points less in respondents with non-health-related qualifications (*p* < 0.001). Race and education as demographic factors were not significant and had no impact on the parsimoniousness of the linear model. To determine whether the fastest responders were influencing a primary outcome in the study, we performed a sensitivity analysis by excluding the top 10% of the fastest responders from the population. When the association between MEDAS score and the three cohorts was examined in the multivariable regression model, all significant findings were also significant when removing 10 % of the fastest responders (data not shown).

**TABLE 2:** Linear regression analysis using an unadjusted and multivariable backward stepwise model to assess MD adherence in the 2018, 2020, and 2022 groups.

					Main effects
		β	SE	*p*-Value*	*p*-value^‡^
**Unadjusted model**					
Group					
	2018^∇^	Ref			
	2020	−0.08	0.18	0.64	
	2022	−0.53	0.17	**0.001**	
**Backward stepwise model**					
Group					**0.02**
	2018^∇^	Ref			
	2020	0.07	0.18	0.67	
	2022	−0.36	0.17	**0.03**	
Sex					**0.001**
	Female^∇^	Ref			
	Male	−0.68	0.18	**< 0.001**	
Age					**< 0.001**
	18–24^∇^	Ref			
	25 ≥	1.35	0.42	**0.001**	
Qualification					**< 0.001**
	Yes^∇^	Ref			
	No	−1.13	0.27	**< 0.001**	

^∇^Ref, reference group used in the model.

*regression coefficient *p*-value.

^‡^Main effects were assessed by ANOVA using a type III sum of squares method.

*p*-values < 0.05 are indicated in bold font.

An unadjusted and multivariable linear regression model adjusting for sex, age, ethnicity, education, health-related qualification, and BMI was used to examine the relationship between MD adherence and class standings (Supplementary Table 2). There was not a statistically significant relationship between MD adherence and class standings both in the unadjusted and adjusted models. Group and sex as demographic factors were significant (*p* = 0.002, *p* < 0.001, respectively) and had an effect on the parsimoniousness of the linear model. The MEDAS score was 0.53 ± 0.17 points less in the 2022 group than in the 2020 group (*p* = 0.002), 0.77 ± 0.22 points less in males than females (*p* < 0.001), and 0.80 ± 0.36 points less in obese respondents (*p* = 0.03). However, age, ethnicity, education, health-related qualification, and BMI were not significant demographic or anthropometric variables.

To gain insight into components of the MEDAS score, individual MD questions were also analyzed using an unadjusted and adjusted model for the demographic and anthropometric variables of sex, age, ethnicity, education, health-related qualification, and BMI. As shown in Supplementary Table 3. In the unadjusted model, participants in the 2022 group consumed less olive oil as their primary culinary fat (*p* = 0.03), had more daily servings of butter, margarine, or cream consumption (*p* = 0.02), and had greater weekly consumption frequency of commercial sweets or pastries (*p* = 0.04) compared to 2018 group. In addition, the 2020 group had less weekly wine consumption in glasses (*p* = 0.008) and had less preferential consumption of chicken, turkey, or rabbit meat over veal, pork, hamburger, or sausage (*p* = 0.04) compared to 2018 group. In the adjusted model, both in the 2020 and 2022 groups had lower weekly wine consumption in glasses compared to 2018 group (*p* = 0.002 and *p* = 0.03, respectively).

### 3.3 Perceived barriers to consuming a MD by university students

To evaluate the degree of internal consistency of barrier factor questions in the current population Cronbach’s alpha was determined. Eighteen questions were divided into four categories—Knowledge, Convenience, Sensory Appeal, and Health— to evaluate internal consistency. Values more than 0.70 are considered optimal for determining internal validity, whereas values greater than 0.60 are deemed sufficient (24). Table 3 demonstrates that the Knowledge barrier had a Cronbach’s alpha = 0.31, which is suggesting low reliability for the questions. The reliability of the knowledge barrier was not improved by eliminating specific questions (data not shown). Acceptable reliability was demonstrated by the Convenience (Cronbach’s = 0.68), Sensory Appeal (Cronbach’s = 0.68), and Health barriers (Cronbach’s = 0.83). These findings are consistent with a prior study in a more diverse cohort of participants (15).

**TABLE 3:** Unadjusted and adjusted linear analysis of perceived MD barriers.

	Unadjusted^†^			Adjusted^††^		
**Barrier**	**β**	**SE**	*P*-value*	**β**	**SE**	*P*-value*
**Knowledge (n = 5)^‡^** **(Cronbach’s alpha = 0.31)**						
2018^∇^	Ref			Ref		
2020	−1.25	0.26	< **0.001**	−1.15	0.26	< **0.001**
2022	−1.38	0.24	< **0.001**	−1.33	0.25	< **0.001**
**Convenience (n = 6)^‡^** **(Cronbach’s alpha = 0.68)**						
2018^∇^	Ref			Ref		
2020	−0.27	0.36	0.46	−0.15	0.37	0.69
2022	−0.56	0.34	0.10	−0.34	0.35	0.32
**Sensory Appeal (n = 3)^‡^** **(Cronbach’s alpha = 0.68)**						
2018^∇^	Ref			Ref		
2020	−0.37	0.24	0.13	−0.14	0.25	0.56
2022	−0.36	0.23	0.11	−0.15	0.23	0.52
**Health (n = 4)^‡^** **(Cronbach’s alpha = 0.83)**						
2018^∇^	Ref			Ref		
2020	−0.68	0.32	**0.03**	−0.34	0.32	0.29
2022	−3.65	0.29	< **0.001**	−3.36	0.30	< **0.001**

^‡^Number of questions in each factor.

*p-values < 0.05 are indicated in bold.

^†^Unadjusted linear model.

^††^Adjusted linear model for sex, age, ethnicity, education, and BMI.

^∇^2018 was used as the reference (Ref) group in the linear model.

With the 2018 group as a reference, we assessed Knowledge, Convenience, Sensory Appeal, and Health barriers in the 2020 and 2022 groups using both an unadjusted and an adjusted linear regression model for sex, age, ethnicity, education, health-related qualifications, and BMI. In the unadjusted model, the 2020 and 2022 groups perceived less MD Knowledge barriers (Knowledge: *p* < 0.001 and *p* < 0.001, respectively), and this relationship persisted in the adjusted model (Knowledge: *p* < 0.001 and *p* < 0.001, respectively). Additionally, the 2020 and 2022 groups perceived less MD health barriers in the unadjusted model (Health: *p* = 0.03 and *p* < 0.001, respectively), and this correlation maintained in the adjusted model in the 2022 group (Health: *p* < 0.001).

### 3.4 Perceived benefits to consuming a MD by university student

The perceived benefits of consuming an MD among respondents were evaluated using characteristics related to Mood, Sensory Appeal, Price, Familiarity, Natural Content, Ethical Concerns, Weight Loss, and Health. Internal validity was assessed by Cronbach’s alpha and was found to be acceptable for each factor (Mood = 0.86, Sensory Appeal = 0.86, Price = 0.54, Familiarity = 0.72, Natural Content = 0.89, Ethical Concerns = 0.86, Weight Loss = 0.87 and Health = 0.96). These findings are consistent with a prior study in a more diverse cohort of participants (15).

To evaluate the benefits of adopting an MD in the 2020 and 2022 groups using the 2018 group as a reference, a linear regression model that was unadjusted, or adjusted for age, BMI, sex, education, health-related qualifications, and ethnicity was utilized (Table 4). In both the unadjusted and adjusted models, the 2020 group perceived the MD to have greater: (1) Health benefits (unadjusted *p* = 0.005, adjusted *p* = 0.004); (2) Weight Loss (unadjusted: Weight Loss: *p* = 0.009, adjusted: Weight Loss: *p* = 0.03); (3) Ethical Concern benefits (unadjusted *p* = 0.009, adjusted *p* = 0.015); (4) Natural Content benefits (unadjusted *p* = 0.003, adjusted *p* = 0.006); and (5) Sensory Appeal benefits (unadjusted *p* = 0.003, adjusted *p* = 0.001).

**TABLE 4:** Unadjusted and adjusted linear analysis of perceived MD benefits.

	Unadjusted^†^			Adjusted^††^		
**Benefits**	**β**	**SE**	*P*-value*	**β**	**SE**	*P*-value*
**Health (n = 10)^‡^ (Cronbach’s alpha = 0.96)**						
2018^∇^	Ref			Ref		
2020	1.78	0.64	**0.005**	1.89	0.66	**0.004**
2022	−16.54	0.59	**< 0.001**	−16.32	0.61	**< 0.001**
**Weight loss (n = 2) (Cronbach’s alpha = 0.87)**						
2018^∇^	Ref			Ref		
2020	0.38	0.14	**0.009**	0.32	0.15	**0.03**
2022	−3.32	0.13	**< 0.001**	−3.36	0.14	**< 0.001**
**Ethical (n = 2) (Cronbach’s alpha = 0.86)**						
2018^∇^	Ref			Ref		
2020	0.41	0.17	**0.009**	0.41	0.17	**0.015**
2022	−2	0.16	**< 0.001**	−2.03	0.16	**< 0.001**
**Natural Content (n = 2) (Cronbach’s alpha = 0.89)**						
2018^∇^	Ref			Ref		
2020	0.44	0.15	**0.003**	0.42	0.15	**0.006**
2022	−3.83	0.14	**< 0.001**	−3.82	0.14	**< 0.001**
**Familiarity (n = 3) (Cronbach’s alpha = 0.72)**						
2018^∇^	Ref			Ref		
2020	0.35	0.25	0.15	0.36	0.26	0.16
2022	−1.39	0.23	**< 0.001**	−1.42	0.24	**< 0.001**
**Price (n = 2) (Cronbach’s alpha = 0.54)**						
2018^∇^	Ref			Ref		
2020	0.25	0.16	0.12	0.21	0.16	0.19
2022	−0.43	0.15	**0.004**	−0.42	0.15	**0.006**
**Sensory Appeal (n = 2) (Cronbach’s alpha = 0.86)**						
2018^∇^	Ref			Ref		
2020	0.47	0.16	**0.003**	0.53	0.16	**0.001**
2022	−3.23	0.14	**< 0.001**	−3.17	0.15	**< 0.001**
**Mood (n = 3) (Cronbach’s alpha = 0.86)**						
2018^∇^	Ref			Ref		
2020	0.37	0.25	0.13	0.42	0.26	0.10
2022	−3.78	0.23	**< 0.001**	−3.7	0.24	**< 0.001**

^‡^Number of questions in each factor.

*p-values < 0.05 from are indicated in bold font.

^†^Unadjusted linear model.

^††^Adjusted linear model for sex, age, ethnicity, education, and BMI.

^∇^2018 was used as the reference (Ref) group in the linear model.

In contrast, the 2022 group perceived MD to have less: (1) Health Benefits (unadjusted: Health: *p* < 0.001, adjusted: Health: *p* < 0.001); (2) Weight Loss (unadjusted: *p* < 0.001, adjusted: *p* < 0.001); (3) Ethical Concerns (unadjusted: *p* < 0.001, adjusted: *p* < 0.001); (4) Natural Content (unadjusted: *p* < 0.001, adjusted: *p* < 0.001); and (5) Sensory Appeal (Unadjusted: *p* < 0.001, adjusted: *p* < 0.001) in both models.

The benefits of the MD were perceived to be less in the 2022 group both in the adjusted and unadjusted models for: (1) Familiarity (Unadjusted: *p* < 0.001, adjusted: *p* < 0.001); (2) Price (Unadjusted: *p* = 0.004, adjusted: *p* = 0.006); and (3) Mood (Unadjusted: Mood: *p* < 0.001, adjusted: Mood: *p* < 0.001). Familiarity, Price, and Mood as perceived benefits were not significant in the 2020 group in both unadjusted and adjusted models.

### 3.5 Stages of change and demographic influences by university students

We next assessed whether there were differences across the three groups in the stages of change associated with the PAPM (8, 25). We observed significant differences between the groups for 2018, 2020, and 2022 in terms of how participants were distributed according to change stages (*p* < 0.001) (Table 5). Compared to the 2020 group and the 2022 group, the 2018 group had fewer participants in the Unaware/Unengaged category (*p* < 0.001). However, the 2020 group had more than the 2022 group. In addition, the 2018 group had more respondents in the Deciding (*p* < 0.01) and Action/Maintenance categories (*p* < 0.01). The percentages of participants in the Decided Yes and Decided No categories did not differ between groups.

**TABLE 5:** Percent of participants in the 2018, 2020 and 2022 groups by stage of change.

Stages of change*	2018	2020	2022
Unaware/unengaged*	48.8	70.8	68.7
Deciding*	28.7	17.1	20.6
Decided no	4.3	2.3	2.7
Decided yes	10.2	6.9	5.8
Action/maintenance*	7.9	2.8	2.1

*Significance across score categories by Pearson’s chi-squared test (*p* < 0.05).

The probability of being in each stage of change toward adopting the MD was examined using logistic regression to identify the impact of demographic and anthropometric variables (Table 6). If participants were in the 2020 and 2022 groups, they had a statistically significant increased likelihood of being in the Unaware/Unengaged stage (*p* < 0.001, and *p* < 0.001, respectively). Additionally, the Unaware/Unengaged stage was significantly more prevalent among participants aged 25 ≥ (*p* < 0.01), participants with Bachelor’s or Higher Degree (*p* < 0.05) and participants with non-health-related qualification (*p* < 0.001).

**TABLE 6:** Backward stepwise elimination logistic regression of stage of change by demographic factors.

		Stages of change			
	**Unaware/unengaged**	**Deciding**	**Decided yes**	**Decided no**	**Action/maintenance**
	**OR (95% CI)**	**OR (95% CI)**	**OR (95% CI)**	**OR (95% CI)**	**OR (95% CI)**
**Cohort**					
2020	2.14 (1.43–3.23)***	–	–	–	0.32 (0.11–0.78)*
2022	1.87 (1.29–2.70)***	–	–	–	0.25 (0.10–0.62)**
**Sex**					
Male	–	–	–	2.18 (0.82–5.22)	0.24 (0.04–0.85)
**Age**					
25 ≤	0.24 (0.08–0.66)**	–	–	–	5.60 (1.17–20.78)*
**Ethnicity**					
Black	2.57 (0.97–7.59)	–	–	–	–
Other	–	–	–	–	–
**Education**					
Bachelor’s degree or higher	0.33 (0.12–0.83)*	3.62 (1.72–7.69)***	–	–	–
Technical or trade certificate	–	–	–	17.57 (0.65–477.64)	–
**Qualification**					
No	1.58 (2.52–10.08)***	0.52 (0.30–1.04)*	0.23 (0.12–0.47)***	–	–
**BMI**					
Underweight	–	–	–	–	–
Overweight	–	–	–	–	–

*p-value < 0.05.

**p-value < 0.01.

***p-value < 0.001.

–Not applicable.

Regarding the Deciding stage, participants with a Bachelor’s or Higher Degree had greater odds of being in this stage (*p* < 0.001). However, participants without health-related qualification had a lower odd of being in the Deciding stage and in the Deciding Yes stage. Furthermore, there were no significant relationships found for the Decided No group. Lastly, in terms of the Action/Maintenance stage, respondents in the 2020 (*p* < 0.05) and 2022 (*p* < 0.01) groups had a statistically significant lower odds of being in this stage. Participants aged 25 ≥ were more likely to be in the Action/Maintenance stage (*p* < 0.05).

### 3.6 Predictions by demographic factor by university students

We next used logistic regression to examine the prediction of a Low MEDAS score in relation to demographic characteristics. As indicated in Table 7, participants in the 2022 group had a statistically significant increased likelihood of having Low MEDAS (*p* < 0.001). In addition, Low MEDAS was significantly more prevalent among Males (*p* < 0.05), and participants with non-health-related qualification (*p* < 0.001). However, participants aged 25 ≥ had a statistically significant lower odds of having a Low MEDAS score (*p* < 0.05).

**TABLE 7:** Backward stepwise logistic regression of predictions by demographic factors.

	Low Med Score
	**OR (95% CI)**
**Cohort**	
2022	1.93 (1.37–2.74)***
**Sex**	
Male	1.65 (1.05–2.64)*
**Age**	
25 ≤	0.38 (0.15–0.94)*
**Qualification**	
No	2.63 (1.50–4.63)***

*p-value < 0.05.

***p-value < 0.001.

### 3.7 COVID-19 related changes in eating habits by university students

Two of our groups (2020 and 2022) completed the survey instrument during the COVID-19 pandemic. In these two groups, we sought to examine whether there were any changes in eating behavior change. We determined using exploratory factor analysis that the six questions fit a two-factor model. The RMSR was 0.02, which is considered acceptable. The RMSEA was 0.037: this shows a good model fit as it is below 0.05. Finally, the Tucker-Lewis Index (TLI) was 0.962: this is an acceptable value considering its over 0.9. We also assessed the loadings. We found that for Factor 1 (eating habits, portion sizes, and types of foods) all loadings were greater than 0.4 which indicates strong loading. However, for Factor 2 only the eating out question had a loading greater than 0.4 while the other questions on frequency snacking (0.368) and MD types of foods (0.120) indicated weak loading. The internal consistency of the factor questions was then assessed: Factor 1 questions (eating habits, portion sizes, and types of foods) had a Cronbach’s alpha of 0.66 while the Factor 2 questions (eating out, frequency snacking, and MD types of foods) had a Cronbach’s alpha of 0.25 (Table 8). Thus, the Factor 2 questions lack internal consistency and appear to be assessing different behaviors. An unadjusted and adjusted model for the demographic and anthropometric variables of sex, age, ethnicity, education, health-related qualification, and BMI was used to assess the individual eating behavior change questions between the 2020 and 2022 groups. As shown in Table 8, in the unadjusted and adjusted model participants in the 2022 group had a greater frequency of snacking (unadjusted *p* = 0.04, adjusted *p* = 0.02) and a lower frequency of eating out (unadjusted *p* = 0.002, adjusted *p* = 0.003) compared to 2020 group.

**TABLE 8:** Linear regression analysis using an unadjusted and adjusted model to assess eating behavior change questions in the 2020 and 2022 groups.

			β	SE	*p*-value*
**Unadjusted model**					
Factor 1^†^	Change in current eating habits				
		2020^∇^	Ref		
		2022	0.01	0.08	0.84
	Change in current portion sizes				
		2020^∇^	Ref		
		2022	−0.01	0.08	0.87
	Change in types of foods consumed				
		2020^∇^	Ref		
		2022	0.11	0.08	0.18
Factor 2^†^	Change in frequency of snacking				
		2020^∇^	Ref		
		2022	0.24	0.12	**0.04**
	Change in frequency of eating out				
		2020^∇^	Ref		
		2022	−0.36	0.12	**0.002**
	Increase in consumption of MD type foods				
		2020^∇^	Ref		
		2022	0.02	0.06	0.74
**Adjusted model**					
Factor 1^†^	Change in current eating habits				
		2020^∇^	Ref		
		2022	0.01	0.08	0.89
	Change in current portion sizes				
		2020^∇^	Ref		
		2022	−0.02	0.08	0.81
	Change in types of foods consumed				
		2020^∇^	Ref		
		2022	0.16	0.09	0.07
Factor 2^†^	Change in frequency of snacking				
		2020^∇^	Ref		
		2022	0.27	0.12	**0.02**
	Change in frequency of eating out				
		2020^∇^	Ref		
		2022	−0.36	0.12	**0.003**
	Increase in consumption of MD type foods				
		2020^∇^	Ref		
		2022	0.001	0.07	0.98

^†^Factor 1 Cronbach’s alpha = 0.66; Factor 2 Cronbach’s alpha = 0.25.

^∇^2020 was used as the reference (Ref) group in the linear model.

*regression coefficient *p*-value. *p*-values < 0.05 are indicated in bold font.

## 4 Discussion

In contrast to countries in which the MD is a cultural heritage (18, 26–28), there is paucity of research on MD adherence and associated factors impacting adherence among university students in the US. Furthermore, it is not known whether university students’ adoption of a MD dietary pattern has increased over the past six years (2018–2023) since the MD was first named as the healthiest way to eat by the US News and World Report (13). Thus, we employed a recently created survey tool to measure university students’ MD adherence, stage of change toward integrating the MD into their lifestyle, and perceived benefits and barriers to consuming an MD (15).

Our analysis showed that there was neither an increase nor decrease in MEDAS score (adherence to the MD) in the 2020 group, compared to the 2018 group. However, we observed that the 2022 group had a lower MEDAS score compared to the 2018 group. Further, we observed that the consumption of olive oil (MEDAS question 2) was lower while the consumption of butter, margarine, or cream (MEDAS question 6) was greater in the 2022 group compared to the 2018 group. These findings suggest a shift to less healthy fats in the 2022 group compared to the 2018 group. Consistent with the findings on MEDAS scores, we observed that a larger proportion of participants in the 2022 group were in the Unaware/Unengaged stage of change compared to the 2018 group. Our findings are consistent with a nutritional transition occurring globally to a greater consumption of ultra-processed foods which has been hypothesized to be linked to industrialization and technological advances of food systems and the globalization of those systems (29). Interestingly, it was recently reported that 59% of a mostly college educated population either had not heard of or new little about ultra-processed foods (30). Whether raising the awareness of the relationship between ultra-processed foods and obesity and chronic disease risk will increase consumption of a healthy dietary pattern like the MD requires testing.

Contrary to our hypothesis that MD adherence will increase over time, our findings show that MD adherence was actually lower over the six year period in which the MD was named the healthiest eating pattern by the US News and World Report (13). Our findings MD adherence over time is consistent with findings that the obesogenic environment found in almost all high-income countries is increasing (29). Indeed, the southeast US has a high concentration of obesogenic counties (31).

The 2020 and 2022 groups were surveyed early and late, respectively, during the COVID-19 pandemic. It is important to note that the students surveyed in 2022 had 2+ years of living through the pandemic. It has been reported that both high school and university students have been experiencing heightened levels of stress during the COVID-19 pandemic (32–34). This stress has affected individuals’ food choices, with positive and negative emotions, leading them to prefer less healthy, more palatable, and energy-dense options during the COVID-19 pandemic (11). It is possible that the impact of stress and emotional eating on dietary behaviors during the COVID-19 pandemic could be a contributing factor to the decreased adherence to a Mediterranean-style diet among the 2022 group, particularly the length of the time in which the 2022 participants lived through the pandemic. Based on our examination of the individual questions regarding changes in eating behavior, it was found that greater snacking and less frequent eating out was associated with the 2022 group. This outcome aligns with findings on the general population, which have indicated an increase in snacking patterns and habits during COVID-19 pandemic (35–37).

In our study, we observed that individuals with a bachelor’s degree or higher were more than four times as likely to be in the “Deciding” stage and less likely to be in the “Unaware/Unengaged” stage, while those with a technical or trade certificate were approximately twenty times more likely to be in the “Decided No” category. Consistent with these findings, participants in the age group of ≥25 were found to be more than 8 times as likely to be in the “Action/Maintenance” stage of MD adherence, and less likely to be in the “Unaware/Unengaged” stage. Taken together, these results are in agreement with prior findings demonstrating a significant relationship between education level and MD adherence in populations from both countries with and without a cultural heritage of the MD (20, 38–41). MD adherence also increased with student age (18, 42). Indeed, it has been reported that older generations are more likely to adhere to traditional diets, while younger generations tend to adopt more Western-style diets (43, 44).

In all three survey periods the majority of participants were female, which is consistent with previous studies with university students (45, 46). We observed that females obtained significantly higher MEDAS scores compared to males. The results are consistent with previous studies conducted in the Mediterranean region which found that women, both in the general population and in medical students, had a greater tendency to follow the MD compared to men (47, 48). Similarly, in the United States, females were more likely to have a high score for a Mediterranean-style dietary pattern (49). It has been observed that individuals who follow the MD more closely tend to have a lower likelihood of being overweight or obese (50–52). Consistent with these findings, we observed that individuals who were classified as obese had a lower score on the MEDAS score.

In both the unadjusted and adjusted models, we found that the Knowledge barrier to adopting the MD was perceived significantly less in the 2020 and 2022 groups compared to the 2018 group. Knowledge can act as an obstacle, impeding individuals from making healthier food choices, or as an asset, aiding them in making informed decisions about their diet (39, 53). We also observed that the Health barrier was perceived significantly less in the 2022 group compared to the 2018 group in both the unadjusted and adjusted models. In contrast to these findings, the 2022 group perceived all eight benefits of the MD (Health, Weight Loss, Ethical Concern, Natural Content, Familiarity, Price, Sensory Appeal, and Mood) to be less beneficial. Interestingly though, all eight perceived benefits of the MD were significantly greater in the 2020 group compared to the 2018 group. Thus, the perceived benefits to consuming a MD were not consistent over time, and our findings indicate that the 2022 participants were less aware of the benefits to consuming a MD. The 2022 participants’ findings in which we observed lower MD adherence scores and perceived benefits of the MD is consistent with our previous results indicating that participants in the Stroke Belt had both lower MD adherence scores and did not perceive Price or Familiarity to be benefits of the MD (15). It is not known whether the longitudinal changes in perceived benefits of the MD were confounded by the COVID-19 pandemic or whether participants will continue with being less aware of the MD benefits. Future studies should be conducted to address this question. To mitigate the long-term impact of not being aware of the MD benefits targeted interventions and education are needed. A potential policy action that universities can undertake to promote healthy diet patterns which is a key component of wellbeing and health promotion is to adopt the Okanagan Charter: An International Charter for Health Promoting Universities & Colleges (54).

A limitation of the present study is that it was conducted solely among students enrolled in the introductory nutrition course, and it is worth noting that the majority of participants in the study were females. Future studies should be conducted to address whether our findings are generalizable to men, and to students in different regions of the US. Moreover, data on stage of change and adherence to the MD were self-reported, which could have been influenced by personal biases or self-selection bias. Finally, the self-reported data, including weight, height, and dietary assessment, may not accurately reflect actual values. This is because participants may have over- or under-reported their weight or height, or had difficulty accurately recalling and reporting their dietary intake, which could have resulted in measurement error and affected the overall findings of the study. Additionally, some participants may have intentionally provided inaccurate information, further impacting the reliability of the data. A major limitation of our COVID-19-related questions is that we did not assess test-retest reliability. However, factor analysis revealed a good fit for a two-factor model. A strength of the current study is that we surveyed participants three times over the course of six years using validated survey questions to gain an understanding of trends in MD adherence and the perceived barriers and benefits to consuming a MD. In addition, to minimize confounding variables, we surveyed the same university course at the same point in the academic year.

In summary, more participants in the 2022 group were categorized in the Unaware/Unengaged stage of change to adopting a MD, and the MD adherence scores in the 2022 group were lower compared to the 2018 group. Even though the 2022 group perceived less barriers to MD consumption compared to the 2018 group, the 2022 group perceived fewer benefits of the MD compared to the 2018 group. Education and awareness-raising about the benefits of the MD may be important in increasing adherence among this group. However, whether the lack of knowledge on the health benefits to consuming a MD played a role in MD adherence will require further examination. Given our findings on MD adherence in the 2022 group, further studies are required to examine whether the influence of the COVID-19 pandemic and length of time the participants experienced the COVID-19 pandemic played a role in MD adherence. Regardless, there is a critical need to promote healthy diet patterns such as the MD.

## Data availability statement

The datasets presented in this study can be found in online repositories. The names of the repository/repositories and accession number(s) can be found below: Harvard Dataverse repository https://doi.org/10.7910/DVN/GL4VHC.

## Ethics statement

The studies involving humans were approved by the Institutional Review Board of Auburn University. The studies were conducted in accordance with the local legislation and institutional requirements. The participants provided their written informed consent to participate in this study.

## Author contributions

SY: Data curation, Formal analysis, Investigation, Methodology, Writing – original draft, Writing – review & editing. PD: Formal analysis, Writing – review & editing. CK: Formal analysis, Methodology, Writing – review & editing. AF: Conceptualization, Formal analysis, Methodology, Writing – review & editing. MG: Conceptualization, Data curation, Formal analysis, Funding acquisition, Methodology, Project administration, Supervision, Writing – original draft, Writing – review & editing.

## Footnotes

1
https://riskcalc.org/samplesize/
