# Supplementary material for: Longitudinal changes in Mediterranean diet adherence and perceived benefits and barriers to its consumption in US university students
Source: Front Nutr. 2024 Jul 2;11:1405369. doi: 10.3389/fnut.2024.1405369 (PMC11250372; doi:10.3389/fnut.2024.1405369)
Supplement: Supplementary file 1 [file Data_Sheet_1.pdf]

We would like to ask you a few questions about your diet	Check the box that applies		
1. Do you use olive oil as main culinary fat?	Yes ☐	No ☐	
2. How many tablespoons of olive oil do you consume in a given day (including oil used for frying, salads, out-of-house meals, etc.)?	<1 ☐	1-4 ☐	>4 ☐
3. How many vegetable servings do you consume per day? (1 serving: ½ cup cooked, 1 cup raw [consider side dishes as half a serving])	<1 ☐	1-2 ☐	>2 ☐
4. How many fruit units (including natural fruit juices) do you consume per day? (1 serving: 1 cup)	<1 ☐	1-3 ☐	>3 ☐
5. How many servings of red meat, hamburger, or meat products (ham, sausage, etc.) do you consume per day? (1 serving: 2-3 ounces)	<1 ☐	1-3 ☐	>3 ☐
6. How many servings of butter, margarine, or cream do you consume per day? (1 serving: 1 tablespoon)	<1 ☐	1-3 ☐	>3 ☐
7. How many sweet or carbonated beverages do you drink per day?	<1 ☐	1-3 ☐	>3 ☐
8. How many glasses of wine do you drink per week? ☐ Red ☐White ☐Both	<2 ☐	2-7 ☐	>7 ☐
9. How many servings of legumes (beans, black eyed peas) do you consume per week? (1 serving: 1 cup)	<1 ☐	1-3 ☐	>3 ☐
10. How many servings of fish or shellfish do you consume per week? (1 serving: 2-3 ounces of fish or 3 ounces of shellfish)	<1 ☐	1-3 ☐	>3 ☐
11. How many times per week do you consume commercial sweets or pastries (not homemade), such as cakes, cookies, biscuits, or custard?	<3 ☐	3-5 ☐	>5 ☐
12. How many servings of nuts (including peanuts) do you consume per week? (1 serving: ¼ cup)	<1 ☐	1-3 ☐	>3 ☐
13. Do you preferentially consume chicken, turkey, or rabbit meat instead of veal, pork, hamburger, or sausage? Are you a vegetarian or vegan? ☐Yes ☐No	Yes ☐	No ☐	
14. How many times per week do you consume boiled vegetables, pasta, rice, or other dishes with a sauce of tomato, garlic, onion, or leeks without meat sautéed in olive oil?	<1 ☐	1-2 ☐	>2 ☐

The next set of questions and responses are based on your knowledge, attitudes, and beliefs about a Mediterranean-based diet.

In this survey a Mediterranean-based diet is characterized by a high intake of fruit, vegetables, olive oil, nuts, and cereals; a moderate intake of fish and poultry; a low intake of dairy products, red meat, processed meats, and sweets; and wine in moderation, consumed with meals.

Check the box that applies.

1. Have you ever heard of about a Mediterranean-based diet?
   - D Yes [if you checked Yes, go to Question 2]
   - D No
2. Are you currently eating a Mediterranean-based diet?
   - D Yes
   - D No [if you checked No, go to Question 3]
3. Which best describes your thoughts about eating a Mediterranean-based diet?
   - D I've never thought about it.
   - D I'm undecided about it.
   - D I've decided I don't want to eat it.
   - D I've decided I do want to eat it.

For next set of questions check the box to indicate whether you agree or disagree with the statements below.

		Strongly Disagree	Disagree	Neither Agree or Disagree	Agree	Strongly Agree
4.	I need more information about a Mediterranean-based diet.	D	D	D	D	D
5.	I do not think about the nutritional aspects of the types of foods I eat.	D	D	D	D	D
6.	I find there are a lot of conflicting messages concerning healthy eating.	D	D	D	D	D
7.	It would be too expensive to eat Mediterranean-based diet foods.	D	D	D	D	D
8.	My family/partner won't eat a Mediterranean-based diet.	D	D	D	D	D
9.	Mediterranean-based diet meals or snacks are not available when I eat out.	D	D	D	D	D
10.	Someone else decides on most of the foods I eat.	D	D	D	D	D
11.	It takes too long to prepare Mediterranean-based diet meals.	D	D	D	D	D
12.	I don't want to change my eating habit or routine.	D	D	D	D	D
13.	I don't have enough willpower to eat a Mediterranean-based diet.	D	D	D	D	D
14.	I don't know how to prepare Mediterranean-based diet meals.	D	D	D	D	D
15.	A Mediterranean-based diet would not be tasty enough.	D	D	D	D	D
16.	There is not enough protein in a Mediterranean-based diet.	D	D	D	D	D
17.	If I eat a Mediterranean-based diet, it would not be filling enough.	D	D	D	D	D
18.	If I eat a Mediterranean-based diet, I would miss eating lots of junk.	D	D	D	D	D

19.	There is not enough iron in a Mediterranean-based diet.	D	D	D	D	D
20.	If I eat a Mediterranean-based diet, I would be worried about my health.	D	D	D	D	D
21.	If I eat a Mediterranean-based diet, I wouldn't get enough energy or strength.	D	D	D	D	D

For next set of responses, check the box to indicate whether you agree or disagree with the completion of the following sentence:

By eating a Mediterranean-based diet, I will …

		Strongly Disagree	Disagree	Neither Agree or Disagree	Agree	Strongly Agree
22.	Decrease my saturated fat intake	D	D	D	D	D
23.	Improve my digestion	D	D	D	D	D
24.	Be fit	D	D	D	D	D
25.	Have a better quality of life	D	D	D	D	D
26.	Live longer	D	D	D	D	D
27.	Be healthier by decreasing my intake of chemicals, steroids, and antibiotics that are found in meat	D	D	D	D	D
28.	Eat more fruits and vegetables	D	D	D	D	D
29.	Reduce my chances of developing major diseases	D	D	D	D	D
30.	Eat high protein foods	D	D	D	D	D
31.	Eat foods high in fiber and roughage	D	D	D	D	D
32.	Eat foods to help me control my weight	D	D	D	D	D
33.	Use olive oil which is more healthy for me and/or my family	D	D	D	D	D
34.	Help the environment	D	D	D	D	D
35.	Help animal welfare / rights	D	D	D	D	D
36.	Eat foods that contains natural ingredients	D	D	D	D	D
37.	Eat foods that are easy to plan, buy, and prepare	D	D	D	D	D
38.	Eat foods that are familiar	D	D	D	D	D
39.	Eat foods that are like the foods I ate when I was a child	D	D	D	D	D
40.	Save money	D	D	D	D	D
41.	Eat foods that are good value for money	D	D	D	D	D
42.	foods that tastes better than processed foods	D	D	D	D	D
43.	Use olive oil to improve the taste of cooked meals	D	D	D	D	D
44.	Eat a greater variety of foods	D	D	D	D	D
45.	Be more content with myself	D	D	D	D	D
46.	Eat foods to help me cope with stress	D	D	D	D	D
47.	Eat foods to make me feel good	D	D	D	D	D

The next set of questions and responses are based on the impact that the COVID-19 pandemic is having on your current eating habits.

Check the box that applies.

1. Do you think there has been any change in your current eating habits?
    - ◻ Yes there has been a change
    - ◻ No there has not been a change
    - ◻ Not sure
2. Have your portion sizes changed compared to your usual intake?
    - ◻ Yes my portion sizes have increased
    - ◻ Yes my portion sizes have decreased
    - ◻ No there has not been a change
    - ◻ I don't know
3. Have the types of foods you are currently eating changed compared to your usual intake?
    - ◻ Yes they have
    - ◻ No they have not
    - ◻ Not sure
4. Has the frequency of your snacking changed compared to your usual intake?
    - ◻ Yes I am snacking more
    - ◻ Yes I am snacking less
    - ◻ No it has not changed
    - ◻ Not sure
5. Has the amount of times you eat out changed compared to your usual eating out?
    - ◻ Yes I am eating out more
    - ◻ Yes I am eating out less
    - ◻ No there has not been a change
    - ◻ Not sure
6. Have you increased your consumption of Mediterranean diet-type foods as a result of the pandemic?
    - ◻ Yes I've consumed more Mediterranean diet-type foods
    - ◻ No I have not consumed more Mediterranean diet-type foods
    - ◻ Not sure

Finally, we would like to ask you a few questions about yourself

1. Are you male or female?
   a) Male ☐
   b) Female ☐

2. How old are you?
   a) less than 18 ☐
   b) 18-24 ☐
   c) 25-34 ☐
   d) 35-44 ☐
   e) 45-54 ☐
   f) 55-64 ☐
   g) 65-74 ☐
   h) more than 75 ☐

3. What is your classification in college?
   a) Freshman/first-year ☐
   b) Sophomore ☐
   c) Junior ☐
   d) Senior ☐
   e) Graduate student ☐
   f) Unclassified ☐

4. What is your ethnic origin?
   a) White ☐
   b) Black African ☐
   c) Black other ☐
   d) Indian ☐
   e) Pakistani ☐
   f) Chinese ☐
   g) Asian- other ☐
   Please specify:………………………………………
   h) Any other ethnic group ☐
   Please specify:………………………………………

5. What is the highest level of education you have completed?
   a) Elementary school ☐
   b) Middle school ☐
   c) High school diploma ☐
   d) GED ☐
   e) Technical or trade certificate ☐
   f) Associate degree ☐
   g) Bachelor's degree ☐
   h) Master's or professional degree ☐

6. Do you have any health or nutrition related qualifications?
   a) Yes ☐
   Please specify: ………………………………………
   b) No ☐

7. What is your body weight?

Please specify: ………………………………………

8. What is your height?

Please specify: ………………………………………
