# Supplementary material for: Longitudinal changes in Mediterranean diet adherence and perceived benefits and barriers to its consumption in US university students
Source: Front Nutr. 2024 Jul 2;11:1405369. doi: 10.3389/fnut.2024.1405369 (PMC11250372; doi:10.3389/fnut.2024.1405369)
Supplement: Supplementary file 2 [file Data_Sheet_2.pdf]

Supplemental Table 1: Class standings of participants by groups.

	2018†		2020†		2022†		
	n	%	n	%	n	%	P-value
Class							0.1266
Freshman	-	-	67	31.3	75	25.8	
Sophomore	-	-	96	44.9	140	48.1	
Junior	-	-	40	18.7	47	16.2	
Senior	-	-	11	5.1	29	10	

* Significance across score categories by Pearson's chi-squared test
† 2018, 2020, and 2022
- Not applicable

Supplementary Table 2: Linear regression analysis using unadjusted and adjusted model to assess the relationship between MD adherence and class standings.

		β	SE	p-Value*	Main Effects p-Value‡
Unadjusted Model					
Class Standings					0.71
	Freshman	Ref †			
	Sophomore	0.18	0.19	0.34	
	Junior	-0.02	0.25	0.92	
	Senior	0.01	0.33	0.98	
Adjusted Model					
Class Standings					0.54
	Freshman	Ref †			
	Sophomore	0.24	0.19	0.21	
	Junior	0.13	0.26	0.62	
	Senior	0.39	0.35	0.27	
Group					**0.002**
	2020	Ref †			
	2022	-0.53	0.17	**0.002**	
Sex					**<0.001**
	Female	Ref †			
	Male	-0.77	0.22	**<0.001**	
Age					0.90
	18-24	Ref †			
	25 ≤	-0.07	0.58	0.90	
Ethnicity					0.24
	White	Ref †			
	Black	0.85	0.64	0.18	
	Other	0.39	0.36	0.27	
Education					0.88
	High School or lower	Ref †			
	Associate's degree	-0.24	0.38	0.52	
	Bachelor's degree or higher	0.27	0.85	0.75	
	GED	-0.48	0.69	0.48	
	Technical or trade certificate	0.76	1.84	0.68	
Qualification					0.13
	Yes	Ref †			
	No	-0.71	0.47	0.13	
BMI					0.10
	Healthy	Ref †			

Obese	-0.80	0.36	**0.03**
Overweight	-0.26	0.22	0.24
Underweight	0.25	0.38	0.51

† Ref, reference group
* regression coefficient p value
‡ Main effects were assessed by ANOVA using a type III Sum of Squares method
p values < 0.05 are indicated in bold font

Supplemental Table 3: Linear regression analysis using an unadjusted and adjusted model to assess individual MD questions in the 2018, 2020 and 2022 groups.

		β	SE	p-Value*
**Unadjusted Model**				
Olive oil as primary culinary fat				
	2018	Ref †		
	2020	-0.01	0.05	0.78
	2022	-0.09	0.04	**0.03**
Olive oil consumption in tablespoons per day				
	2018	Ref †		
	2020	-0.0005	0.01	0.97
	2022	-0.006	0.01	0.60
Daily vegetable servings consumed				
	2018	Ref †		
	2020	-0.06	0.03	0.06
	2022	-0.05	0.03	0.09
Daily fruit units consumed				
	2018	Ref †		
	2020	0.004	0.03	0.90
	2022	0.03	0.03	0.25
Daily servings of red meat, hamburger, or meat products consumed				
	2018	Ref †		
	2020	0.0007	0.04	0.10
	2022	-0.04	0.04	0.38
Daily servings of butter, margarine, or cream consumed:				
	2018	Ref †		
	2020	-0.07	0.05	0.13
	2022	-0.10	0.04	**0.02**
Daily consumption of sweet or carbonated beverages:				
	2018	Ref †		
	2020	0.04	0.04	0.3
	2022	-0.06	0.04	0.11
Weekly wine consumption in glasses				
	2018	Ref †		

	2020	-0.03	0.01	**0.008**
	2022	-0.02	0.01	0.08
Weekly servings of legumes consumed:				
	2018	Ref †		
	2020	0.03	0.02	0.28
	2022	-0.003	0.02	0.90
Weekly servings of fish or shellfish consumed:				
	2018	Ref †		
	2020	-0.02	0.02	0.34
	2022	-0.02	0.02	0.3
Weekly consumption frequency of commercial sweets or pastries				
	2018	Ref †		
	2020	0.07	0.04	0.12
	2022	-0.08	0.04	**0.04**
Weekly servings of nuts consumed:				
	2018	Ref †		
	2020	0.002	0.03	0.93
	2022	-0.02	0.03	0.34
Preferential consumption of chicken, turkey, or rabbit meat over veal, pork, hamburger, or sausage:				
	2018	Ref †		
	2020	-0.08	0.04	**0.04**
	2022	-0.01	0.04	0.77
Vegetarian or Vegan:				
	2018	Ref †		
	2020	-0.006	0.02	0.72
	2022	-0.01	0.02	0.44
Weekly frequency of consuming boiled vegetables, pasta, rice, or other dishes with a sauce of tomato, garlic, onion, or leeks without meat sautéed in olive oil:				
	2018	Ref †		
	2020	0.03	0.04	0.40
	2022	-0.05	0.04	0.20

**Adjusted Model**				
Olive oil as primary culinary fat				
	2018	Ref †		
	2020	0.02	0.05	0.60
	2022	-0.05	0.04	0.22
Olive oil consumption in tablespoons per day				
	2018	Ref †		
	2020	0.0006	0.01	0.96
	2022	-0.009	0.01	0.49
Daily vegetable servings consumed				
	2018	Ref †		
	2020	-0.05	0.03	0.13
	2022	-0.02	0.03	0.43
Daily fruit units consumed				
	2018	Ref †		
	2020	-0.0001	0.03	0.10
	2022	0.03	0.03	0.25
Daily servings of red meat, hamburger, or meat products consumed				
	2018	Ref †		
	2020	0.04	0.04	0.36
	2022	-0.02	0.04	0.67
Daily servings of butter, margarine, or cream consumed:				
	2018	Ref †		
	2020	-0.05	0.05	0.29
	2022	-0.08	0.04	0.08
Daily consumption of sweet or carbonated beverages:				
	2018	Ref †		
	2020	0.06	0.04	0.20
	2022	-0.05	0.04	0.23
Weekly wine consumption in glasses				
	2018	Ref †		
	2020	-0.03	0.01	**0.002**
	2022	-0.02	0.01	**0.03**
Weekly servings of legumes consumed:				
	2018	Ref †		

	2020	0.03	0.03	0.22
	2022	0.007	0.02	0.78
Weekly servings of fish or shellfish consumed:				
	2018	Ref †		
	2020	-0.02	0.02	0.24
	2022	-0.01	0.02	0.39
Weekly consumption frequency of commercial sweets or pastries				
	2018	Ref †		
	2020	0.09	0.05	0.06
	2022	-0.06	0.04	0.15
Weekly servings of nuts consumed:				
	2018	Ref †		
	2020	0.008	0.03	0.80
	2022	-0.01	0.03	0.57
Preferential consumption of chicken, turkey, or rabbit meat over veal, pork, hamburger, or sausage:				
	2018	Ref †		
	2020	-0.05	0.04	0.18
	2022	-0.01	0.04	0.75
Vegetarian or Vegan:				
	2018	Ref †		
	2020	0.002	0.02	0.89
	2022	0.001	0.02	0.94
Weekly frequency of consuming boiled vegetables, pasta, rice, or other dishes with a sauce of tomato, garlic, onion, or leeks without meat sautéed in olive oil:				
	2018	Ref †		
	2020	0.05	0.04	0.25
	2022	-0.03	0.04	0.46

† Ref, reference group
* regression coefficient p value
p values < 0.05 are indicated in bold font
